# P-1438. A Safety and Immunogenicity Study of Novavax's COVID-Influenza Combination and Stand-alone Influenza Vaccines in Adults 65 years and Older

**DOI:** 10.1093/ofid/ofaf695.1625

**Published:** 2026-01-11

**Authors:** Chijioke Bennett, Katherine Smith, Wayne Woo, Susan Neal, Joyce S Plested, Tim Vincent, Mingzhu Zhu, Shane Cloney-Clark, Bridget Riviers, Miranda R Cai, Pratyusha Kajipet, Zhaohui Cai, Iksung Cho, Raburn M Mallory, Robert Walker

**Affiliations:** Novavax, Gaithersburg, Maryland; Novavax, Inc., Gaithersburg, Maryland; Novavax, Inc., Gaithersburg, Maryland; Novavax, Gaithersburg, Maryland; Novavax, Gaithersburg, Maryland; Novavax, Gaithersburg, Maryland; Novavax, Gaithersburg, Maryland; Novavax, Gaithersburg, Maryland; Novavax, Inc., Gaithersburg, Maryland; Novavax, Inc., Gaithersburg, Maryland; Novavax, Gaithersburg, Maryland; Novavax, Inc., Gaithersburg, Maryland; Novavax, Inc., Gaithersburg, Maryland; Novavax, Inc., Gaithersburg, Maryland; Novavax, Inc., Gaithersburg, Maryland

## Abstract

**Background:**

Using its recombinant protein nanoparticle and Matrix-M^®^ adjuvant technology platform, Novavax has developed both a COVID-19 Influenza Combination vaccine (CIC) and a trivalent nanoparticle influenza hemagglutinin vaccine (tNIV). Here, we report on an ongoing, randomized, observer-blinded trial (CIC-E-301) to evaluate the safety and immunogenicity of these vaccines in adults ≥ 65 years. In CIC-E-301, CIC is compared to Novavax's 2024-2025 COVID-19 vaccine targeting JN.1 (NVX-CoV2705) and Fluzone^®^ High-Dose; tNIV is compared to Fluzone High-Dose.

**Table 1:**
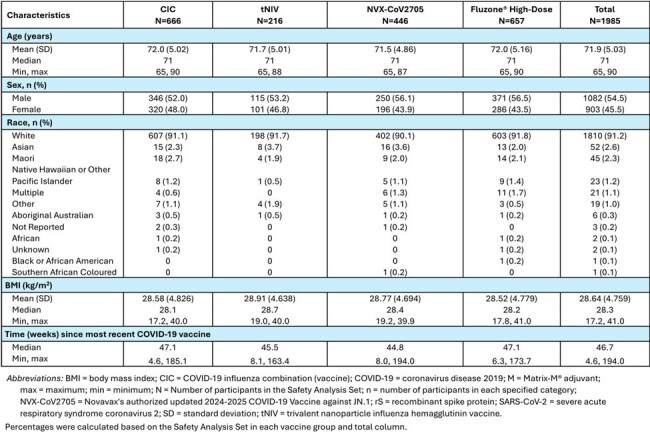
Demographics and Baseline Characteristics for CIC-E-301 – Safety Analysis Set

**Table 2:**
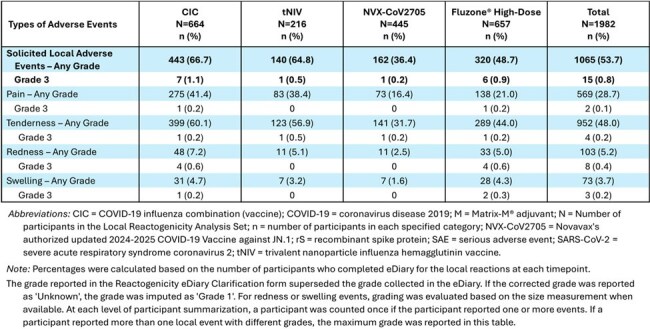
Summary of Solicited Local Injection Site Adverse Events within 7 Days of Vaccination for CIC-E-301 – Local Reactogenicity Analysis Set

**Methods:**

A total of 1985 participants in Australia and New Zealand received a single dose of either CIC, NVX-CoV2705, tNIV, or Fluzone High-Dose in a 3:2:1:3 ratio, respectively. The tolerability and safety of CIC and tNIV were evaluated by assessing reactogenicity for 7 days post dose and unsolicited adverse events (AEs) through Day 28. Immunogenicity was also assessed, with sera collected 28 days post dose and analyzed for vaccine-homologous influenza A and B strain neutralizing antibody (NAb) responses and influenza hemagglutination inhibiting antibodies, and for SARS-CoV-2 NAb responses to the JN.1 strain of SARS-CoV-2. Analyses of data are descriptive, with no prespecified statistical hypotheses.

**Table 3:**
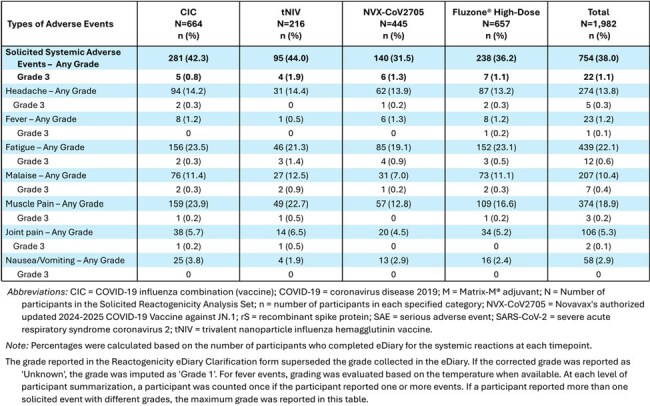
Summary of Solicited Systemic Adverse Events within 7 Days of Vaccination for CIC-E-301 – Systemic Reactogenicity Analysis Set

**Table 4:**
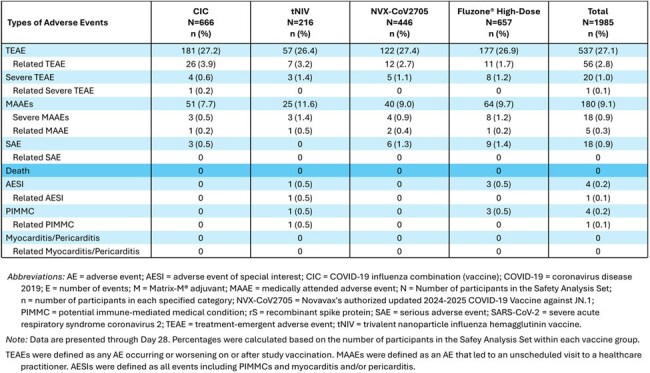
Overall Summary of Unsolicited Adverse Events through Day 28 for CIC-E-301 – Safety Analysis Set

**Results:**

Demographic and baseline characteristics were balanced across the vaccine groups. Overall, the median age of participants was 71 years, 54.5% male/ 45.5% female, 91.2% White, with median time since last COVID-19 vaccination of 46.7 weeks.

A single dose of CIC or tNIV had an acceptable safety and tolerability profile. Solicited local and systemic reactogenicity AEs, respectively, were reported more frequently following CIC (66.7%, 42.3%) and tNIV (64.8%, 44.0%) than NVX-CoV2705 (36.4%, 31.5%) and Fluzone High-Dose (48.7%, 36.2%). Most reactogenicity events in the CIC and tNIV recipients were Grade 1 (∼1% Grade 3) including local tenderness and pain, muscle pain, and fatigue. Unsolicited TEAEs, including related and/or severe TEAEs, MAAEs, and SAEs (reported for 0.5% - 1.4%) were noted at similar frequencies across the vaccine groups.

**Conclusion:**

There were no AESIs reported for CIC or NVX-CoV2705, 1 (0.5%) for tNIV, and 3 (0.5%) for Fluzone High-Dose. There were no events of myocarditis/pericarditis or death.

**Disclosures:**

Chijioke Bennett, MD, MPH, MBA, Novavax, Inc.: employee|Novavax, Inc.: Stocks/Bonds (Public Company) Katherine Smith, M.D., Novavax, Inc.: employee|Novavax, Inc.: Stocks/Bonds (Public Company) Wayne Woo, MS, Novavax, Inc: Employee|Novavax, Inc: Stocks/Bonds (Public Company) Susan Neal, n/a, Novavax Inc.: employee|Novavax Inc.: Stocks/Bonds (Public Company) Joyce S. Plested, n/a, Novavax, Inc.: employee|Novavax, Inc.: Stocks/Bonds (Public Company) Tim Vincent, n/a, Novavax, Inc.: employee|Novavax, Inc.: Stocks/Bonds (Public Company) Mingzhu Zhu, n/a, Novavax, Inc.: employee|Novavax, Inc.: Stocks/Bonds (Public Company) Shane Cloney-Clark, n/a, Novavax, Inc.: employee|Novavax, Inc.: Stocks/Bonds (Public Company) Bridget Riviers, Ph.D., Novavax, Inc: Employee|Novavax, Inc: Stocks/Bonds (Public Company) Miranda R. Cai, PhD, Novavax, Inc.: employee|Novavax, Inc.: Stocks/Bonds (Public Company) Pratyusha Kajipet, M.S., Novavax: employee|Novavax: Stocks/Bonds (Public Company) Zhaohui Cai, PhD, Novavax, Inc.: employee|Novavax, Inc.: Stocks/Bonds (Public Company) Iksung Cho, MS, Novavax, Inc.: employee|Novavax, Inc.: Stocks/Bonds (Public Company) Raburn M. Mallory, M.D., Novavax: I am an employee of Novavax|Novavax: Stocks/Bonds (Public Company) Robert Walker, M.D., Novavax, Inc.: employee|Novavax, Inc.: Stocks/Bonds (Public Company)

